# The Impact of Excision Interval on Equine Melanoma Progression: Time Matters?

**DOI:** 10.3390/ani14081244

**Published:** 2024-04-22

**Authors:** José Pimenta, Justina Prada, Isabel Pires, Mário Cotovio

**Affiliations:** 1 CECAV-Veterinary and Animal Research Center, University of Trás-os-Montes e Alto Douro, 5000-801 Vila Real, Portugal; jprada@utad.pt (J.P.); ipires@utad.pt (I.P.); mcotovio@utad.pt (M.C.); 2 Associate Laboratory for Animal and Veterinary Sciences (AL4AnimalS), 5000-801 Vila Real, Portugal; 3 CIVG–Vasco da Gama Research Center, EUVG–Vasco da Gama University School, 3020-210 Coimbra, Portugal; 4 Veterinary Sciences Department, University of Trás-os-Montes e Alto Douro, 5000-801 Vila Real, Portugal; 5 Faculty of Veterinary Medicine, Lusófona University, Campo Grande 376, 1749-024 Lisbon, Portugal

**Keywords:** equine, melanomas, time, clinical factors, histology

## Abstract

**Simple Summary:**

Equine melanomas are a common problem in clinical practice. Although a known issue in the equestrian world, there is still some lack of knowledge about various aspects of the disease. Due to the misconception that these tumors do not cause problems, there is a tendency to leave them to be treated at a later date. However, there is still little evidence regarding the effect of time on these tumors. This work aimed to study the clinical and histological differences between tumors excised sooner and later. In this retrospective study, information regarding 34 horses and 42 tumors was analyzed. According to the results, tumors with delayed excision were statistically more likely to acquire larger dimensions (*p* = 0.038) and be malignant (*p* = 0.035). Furthermore, delayed excision was also associated with a higher number of tumors (*p* = 0.011), since horses with a tumor for more than 6 years often had multiple tumors. With this study, we conclude that delayed excision has clinical and histological consequences for equine melanoma, highlighting the importance of surgical removed at an early stage, when excision is easier, preventing future complications.

**Abstract:**

Equine melanomas are a common neoplasm in gray horses. However, scientific knowledge about their progression over time is quite scarce. Some owners and veterinarians still believe that early intervention is not necessary, stating that tumors evolve very slowly and intervention could worsen the animal’s condition. This work aims to identify clinical and histological differences that may exist between equine melanomas with different excision intervals (time between tumor detection and surgical excision). A total of 42 tumors (13 benign and 29 malignant) from 34 horses were included in this study. There was a statistically significant association between excision interval and tumor size (*p* = 0.038), with tumors excised later being significantly larger than the ones excised sooner. The excision interval was also statistically associated with the number of tumors (*p* = 0.011), since the horses that carried a tumor for longer seemed to be prone to have multiple tumors. Furthermore, there was an association between excision interval and malignancy (*p* = 0.035), with tumor excised later being fives times more likely to be malignant. This study provides evidence of delayed excision’s effect on the progression of equine melanomas. Additionally, it reinforces the importance of the early excision of these tumors.

## 1. Introduction

In horses, skin oncological diseases are a common problem in clinical practice. Among these, equine melanoma is the third most common type of tumor, accounting for 15% of all cutaneous tumors in this species [1,2,3]. This tumor has no gender predisposition and can occur in horses of all coats and ages [4,5,6,7]. However, gray horses have a higher prevalence, since the hereditable genetic trait responsible for graying is also responsible for the development of melanomas [8,9,10,11]. In addition, although congenital occurrences and development in younger horses have been described, most tumors develop around 6 years of age, when the hair-graying process is generally more pronounced [9,12]. Nevertheless, geriatric gray horses are the most affected; it is estimated that 80% of gray horses that are more than 15 years old will probably develop melanoma [13].

Despite its high incidence, horse melanoma has some characteristics that have led to its neglect for many years, even nowadays, such as the slow and apparently benign progression over the years, which have created the misconception that early intervention is not necessary [14]. This has had consequences both from a clinical and from a research point of view, with the scientific literature about this disease being scarce and often based only on clinical comments and personal experiences. Thus, our knowledge about this disease is still very limited [7,13,15,16]. Many of these tumors are detected by owners during grooming, but due to their initially small dimensions and localization, they usually do not cause performance constraints. Due to this fact, and to their intrinsic history of benignity, some veterinarians only see these cases at a more advanced stage, when tumor effects begin to worry owners [1,17,18]. Nevertheless, due to uncertainties in the literature regarding the proper management practices of this disease, such as the timing of excision, future consequences of excision, and applicable treatments, some veterinarians do not advise early intervention [7,14]. However, there are already some studies that have shown no complicated consequences or risk of recurrence after equine melanoma surgical excision [19,20].

In other species, such as humans and dogs, melanomas are known to be a high-risk disease with a rapid progression. However, the clinical progression in horses is quite different [21,22,23,24]. Equine melanoma is characterized by single or multiple nodules that emerge in typical localizations. They often show a slow growth rate over the years and can often remain dormant for long periods. The main disturbance these tumors cause is their space-occupying nature that, depending on the localization, can cause serious impairment of various physiological functions such as vision, eating, breathing, defecating, and urinating [1,5,7,15,16,25].

Many horses remain with these tumors for years until their owners decide to treat them. Although the exact consequences of this wait have not been well described, according to their personal experiences, several authors are of the opinion that most equine melanomas have a progressive nature that depends on time [7,9,15,26]. In other words, it is thought that, over time, a solitary mass can evolve into multiple tumors, a small tumor can acquire larger dimensions, and an initially benign melanoma can become malignant. However, objective studies that validate these opinions are scarce.

Another distinguishing feature of equine melanomas is that they are less invasive, and metastasis is reported infrequently compared with other species [27]. Furthermore, reported metastases are mainly detected in older horses with advanced stages of the disease [19,28,29,30,31]. Possible causes for this delay in tumor spread have yet to be confirmed [7]. E-cadherin and COX-2 are two biomarkers associated with many tumors’ progression and aggressiveness [32,33,34,35]. E-cadherin is an adhesion molecule, and its loss facilitates tumor cell dissemination [36]. COX-2 is an enzyme involved in carcinogenesis, and its overexpression can promote tumor invasion and metastasis [37,38]. As such, tumor progression is often followed by a loss of E-cadherin and overexpression of COX-2 across time, which promotes tumor cell migration, invasiveness, and metastization [37,39,40]. The relationship between E-cadherin and COX-2 has been established in numerous cancers, where a significant negative correlation was observed and related to tumor progression [34,38,40,41,42].

Considering the scarcity of knowledge about these tumors, this work aims to evaluate the influence of delayed excision on the clinical, histological, and immunohistochemical characteristics of equine melanoma. With this work, we try to understand what evidence-based advice veterinarians may give to owners regarding early intervention on these tumors.

## 2. Materials and Methods

### 2.1. Tissue Samples

Given the retrospective nature of this study, formalin-fixed, paraffin-embedded samples of primary equine melanocytic tumors were used. This study included all the tumors present in the repository, which were collected by field clinicians and sent for histological evaluation between 2010 and 2023, and contained all the clinical information mentioned below. Regarding the exclusion criteria, tumors with incomplete clinical information and tumors that raised doubts during histological classification (benign or malignant) were not included.

### 2.2. Clinical Information

Information regarding the number of tumors per horse (single or multiple), dimension of the analyzed tumor (small: <2 cm; medium: 2–4 cm; and large: >4 cm), and excision interval (EI) (period of time between tumor detection by the owner and surgical excision) was collected. For statistical purposes, excision interval was divided into two groups, according to the median of excision interval: EI < 6 years and EI ≥ 6 years. Although information regarding the number of tumors per horse was collected, for most animals, only one of the excised tumors was histologically and immunohistochemically evaluated. In these cases, the tumor analyzed was the one that, according to the owner, appeared first.

Some general information like age, gender, mass localization, and breed were also collected. Age was divided into geriatric (≥15 years old), adult (6–14 years old), and young (≤5 years old).

### 2.3. Histopathological Evaluation

Tumors were histologically evaluated by two independent pathologists (JP and IP) and classified according to epidermal ulceration (present or absent); encapsulation (present or absent); and cell shape (epithelioid, spindle, or mix).

Tumors were also histologically classified as benign or malignant, following an adaptation of the methodology performed by [43]. The histological features used for this classification were mitotic count (0 mitosis; <10 mitosis; and ≥10 mitosis per ten high power fields (HPF)); nuclear grade (I—minimal variations in shape and size of nuclei; II—moderate alterations on nuclear shape; and III—irregular and larger than normal nuclei), and tumor vascular emboli (present or absent). Mitotic count was divided into absent (0 mitosis) and present (<10 or ≥10 mitosis).

According to these features, a melanoma was considered malignant if contained tumor vascular emboli, more than 10 mitoses in 10 HPF, and a nuclear grade of II or III.

### 2.4. Immunohistochemistry

The immunohistochemical analysis was performed separately for each biomarker (monoclonal anti-COX-2 (Thermo Scientific™ Lab Vision™, clone SP21, Waltham, MA, USA) diluted 1:40 in phosphate-buffered saline (PBS) and monoclonal anti-E-cadherin (4A2C7, Invitrogen, Waltham, MA, USA) diluted 1:50 in PBS) with a commercial detection system (NovoLink Polymer Detection System; Novocastra, Leica Biosystems, Newcastle, UK).

Antigen retrieval (3 cycles of 5 min at 750 W on microwave) was performed with a citrate buffer solution (0.01 M pH 6.0 ± 2). A bleaching protocol was performed after cooling the slides. Endogenous peroxidase and endogenous protein were blocked for 5 min. Both primary antibodies were incubated at 4 °C overnight. After incubation with a secondary antibody, an immunolabeling visualization was performed using 3,3′—diaminobenzidine tetrahydrochloride (DAB) chromogen. Counterstaining was performed with Gill’s hematoxylin.

### 2.5. Immunohistochemical Evaluation

Two independent pathologists (IP and JP) blindly evaluated the immunolabeling semi-quantitatively. Positivity for both biomarkers was indicated by a brown membranous and/or cytoplasmatic labeling. Positive controls were used for E-cadherin (epidermis) and COX-2 (equine kidney) in each staining run, as well as negative controls (omission of primary antibody).

For both biomarkers, the immunohistochemical evaluation was classified for extension as (0) negative; (1) 1–5%; (2) 6–20%; (3) 21–50% of labelled cells; or (4) >50%, and intensity as (0) negative, (1) weak; (2) moderate; or (3) strong. An immunohistochemical score (IHS) was created for each biomarker by multiplying the extension by the labelling intensity, resulting in a value between 0 and 12.

For statistical purposes, we followed an adaptation of the methodology used by [44], in order to divide tumors into three categories: tumors with IHS of 0 were considered negative, IHS ≤ 6 were considered to have a low E-cadherin/COX-2 expression, and tumors with IHS > 6 were considered to have a high E-cadherin/COX-2 expression.

### 2.6. Statistical Analysis

A Shapiro–Wilk test was performed in order to study the distribution of the continuous variable “excision interval”. Since it did not follow a normal distribution, non-parametric tests were used. A Mann–Whitney test was performed on categorical variables with only two groups in order to assess whether there were differences in the median of excision interval between these groups. The Mann–Whitney test was used for the categorical variables “number of tumors” and “histological classification”, in order to find out whether multiple tumors had a higher median of “excision interval” and whether malignant tumors also had a higher median of “excision interval”. A Kruskall–Wallis test was used for the same statistical purpose, but in this case, it was applied to the categorical variable that had three groups (tumor size: small, medium, or large).

In cases where the variable “excision interval” was used as a categorical variable (<6 years or ≥6 years), a Chi-square test and Fisher’s exact test were used to evaluate the association between this variable and the others. When applicable, an odds ratio test was performed in order to assess the probability of a tumor changing clinically or histologically according to the excision interval.

In order to evaluate the correlation between excision interval and E-cadherin/COX-2 expression (both as continuous variables), a Spearman’s test was performed. The same test was used to evaluate the correlation between both biomarkers. All the results were considered significant when *p* < 0.05.

## 3. Results

### 3.1. Clinical Features

The study sample included a total of 42 tumors from 34 horses. The median and interquartile range (IQR) of excision interval was 6 (3.75) years. All of the tumors had been present for more than a year, except for one 2-year-old horse that had had the tumor for 6 months. Regarding excision interval, 27 tumors had an excision interval of ≥6 years and 15 tumors had an interval of <6 years. The five longest-lasting tumors had been present for 10 years. Concerning tumor size, twenty tumors had small dimensions, nine had medium dimensions, and thirteen had large dimensions. Regarding number of tumors, twenty-nine horses had multiple tumors and five horses had only a single tumor.

Regarding gender, 16 horses were males and 18 were females. Of the mares, 7 were for breeding purposes and 11 were not. A total of seventeen horses were purebred Lusitano, fifteen were crossbred, and two were Arabian. Regarding age, twenty-two horses were geriatric, eleven were adults, and one was young. Tumor localizations were distributed around the perianal region (*n* = 25) and tail (*n* = 17).

### 3.2. Influence of Time on Clinical Features

A statistically significant relationship between excision interval and tumor size was obtained (*p* = 0.003). According to the Kruskal–Wallis results, the medians of the excision intervals were significantly higher for larger tumors than for smaller tumors. However, no significant differences were seen between small and medium tumors (*p* = 0.44), nor between medium and large tumors (*p* = 0.231).

**Figure 1:**
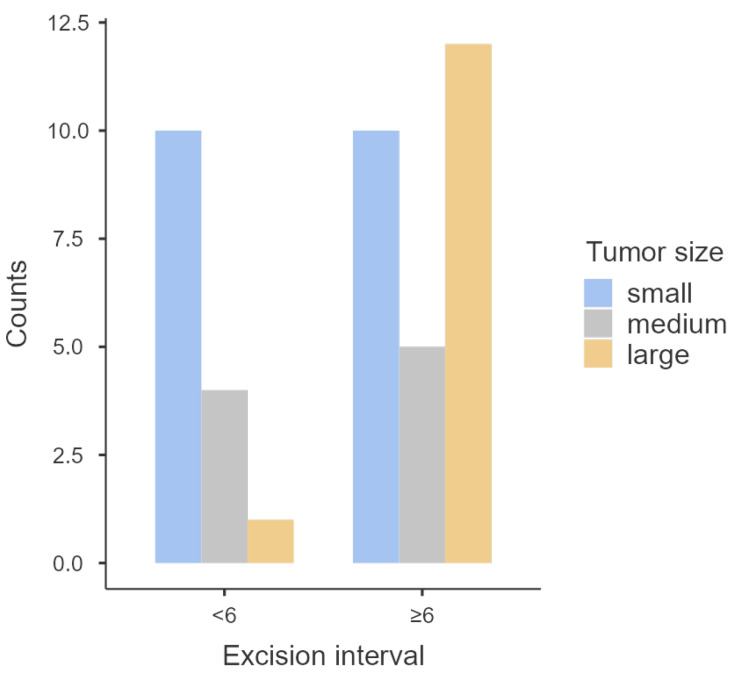
Distribution of different tumor sizes according to different excision intervals.

When dividing excision interval into two classes (≥6 years and <6 years), there was also a significant statistical association with tumor size (*p* = 0.038). Tumors excised later often presented with larger dimensions than tumors excised sooner (Table 1 and Figure 1).

The statistical significance remained (*p* = 0.015) when dividing tumor size into only two categories (small: ≤4 cm and large >4 cm). This allowed us to perform an odds ratio test, which indicated that tumors with higher excision intervals (≥6 years) were 11.2 times more likely to have large dimensions.

A statistically significant relationship was also obtained between tumor excision interval and the number of tumors (*p* = 0.002). The median excision interval was higher for horses with multiple tumors.

**Figure 2:**
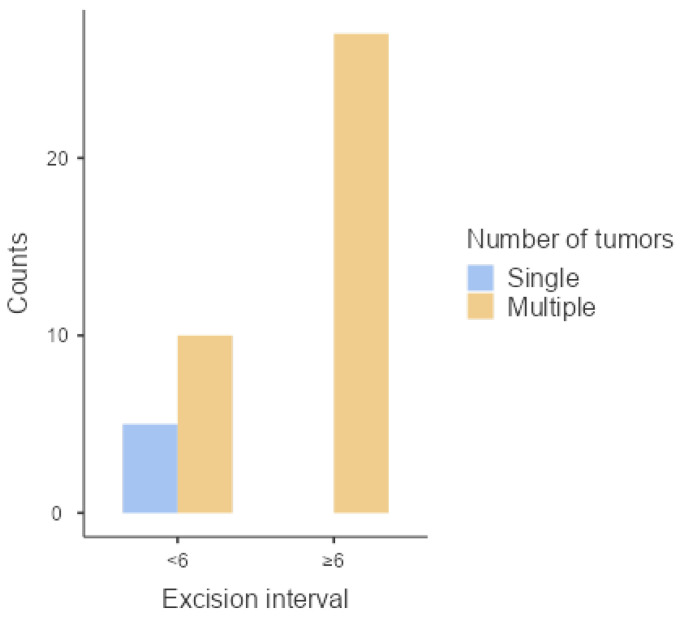
Distribution of number of tumors according to different excision intervals.

When dividing excision interval into two categories (<6 years and ≥6 years), a significant association with the number of tumors (*p* = 0.011) was also obtained, as shown in Table 2 and Figure 2. Horses that have an excision interval equal to or greater than 6 years are 10 times more likely to have multiple tumors.

No association was detected between excision interval and gender (*p* = 1.0) or breed (*p* = 0.56). We also evaluated if there was any difference in excision interval between breeding and non-breeding mares, without any significant result (*p* = 0.15).

Although not related to excision interval, we also evaluated whether tumor size was associated with histological classification or with tumor ulceration, and we did not find any significant result (*p* = 0.72 and *p* = 0.73, respectively).

### 3.3. Histological Features

Of the 42 tumors, 13 were classified as benign and 29 as malignant. All tumors presented mixed cell shapes. Most tumors were well encapsulated (*n* = 26), with some presenting an interruption on the capsule (*n* = 16). Only 10 tumors presented epidermal ulceration, with most tumors having an intact epidermis (*n* = 32). Only eight tumors presented visible mitosis, with six presenting more than ten mitosis and two presenting less than ten mitosis per 10 HPF. A total of 22 tumors presented vascular emboli, and 20 did not. A total of 32 tumors presented an abnormal nuclear grade, and 10 had a normal nuclear grade.

### 3.4. Influence of Time on Histological Features

**Figure 3:**
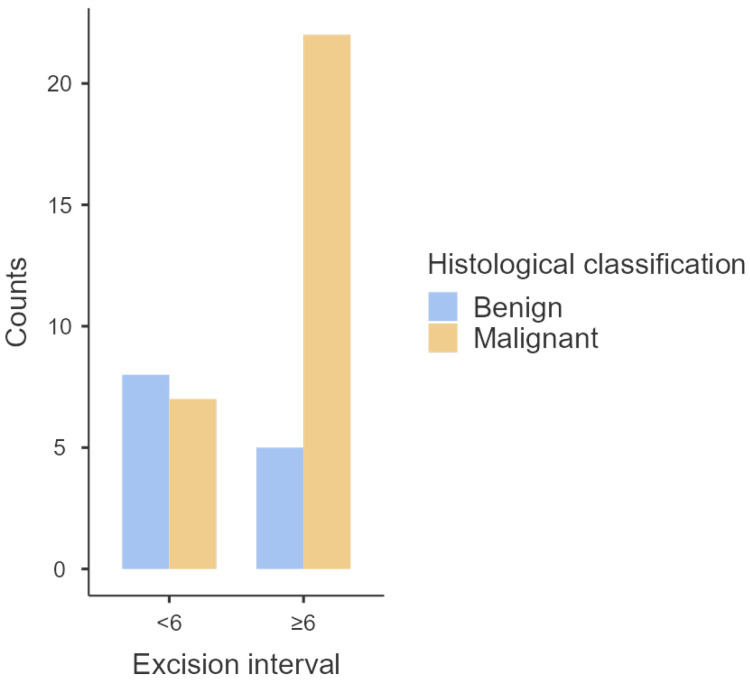
Distribution of histological classification according to different excision intervals.

There was a statistically significant association between histological classification and excision interval (*p* = 0.035), and most tumors with an excision interval of ≥6 years were histologically malignant (Table 3 and Figure 3). Furthermore, according to the odds ratio analysis, tumors with an excision interval of more than 6 years had five times the likelihood of being malignant than tumors with an interval of less than 6 years. According to the Mann–Whitney analysis, malignant tumors also presented significantly higher excision interval medians (*p* = 0.04).

Even though excision interval was associated with histological classification, no association was observed with the individual histological features of malignancy, namely mitotic count (*p* = 0.58), tumor vascular emboli (*p* = 0.74), and nuclear grade (*p* = 0.35).

No association was found between excision interval and tumor encapsulation (*p* = 0.75) or ulceration (*p* = 1.0).

Although unrelated to excision interval, we evaluated whether the presence or absence of epidermal ulceration was associated with histological classification, and we did not find statistically significant results (*p* = 0.70).

### 3.5. Influence of Time on Biomarker Expression

The association and correlation between excision interval and these biomarker expressions were analysed in order to evaluate if E-cadherin and COX-2 expressions are different in tumors excised sooner or later. In order to test the correlation, we used the IHS as a continuous variable (0 to 12). In order to evaluate the association, we used the IHS of the biomarkers divided into three categories (negative, low, and high).

No correlation (*p* = 0.79) was detected between excision interval and E-cadherin score. E-cadherin expression remains practically unchanged over time. A similar result was obtained regarding COX-2 (*p* = 0.72).

**Figure 4:**
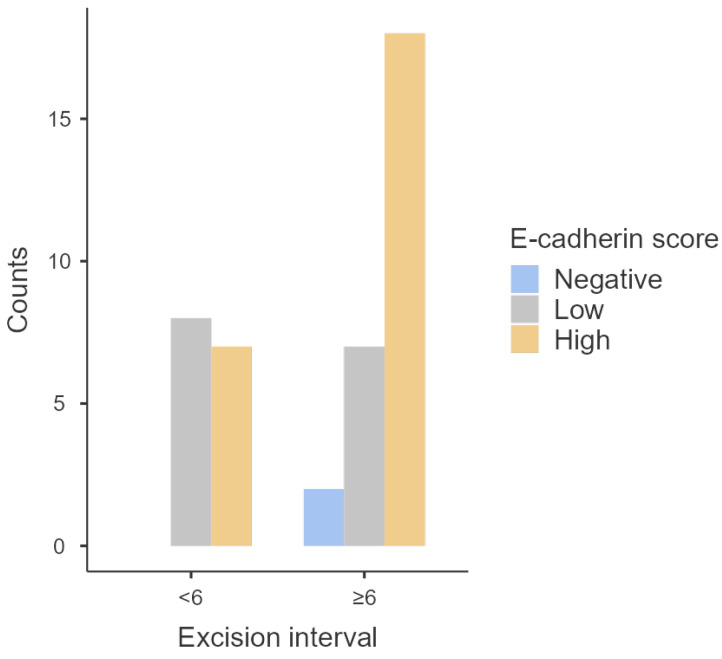
Distribution of E-cadherin scores according to excision intervals.

Regarding E-cadherin, no statistically significant association was found (*p* = 0.20) with excision interval, with most tumors (*n* = 25) maintaining high levels of E-cadherin independent of the time of excision (Table 4 and Figure 4). However, the only two tumors negative for E-cadherin had an excision interval time of more than 6 years. Furthermore, no significant statistical differences were detected in the medians of E-cadherin scores between tumors excised sooner and later (*p* = 0.66).

**Figure 5:**
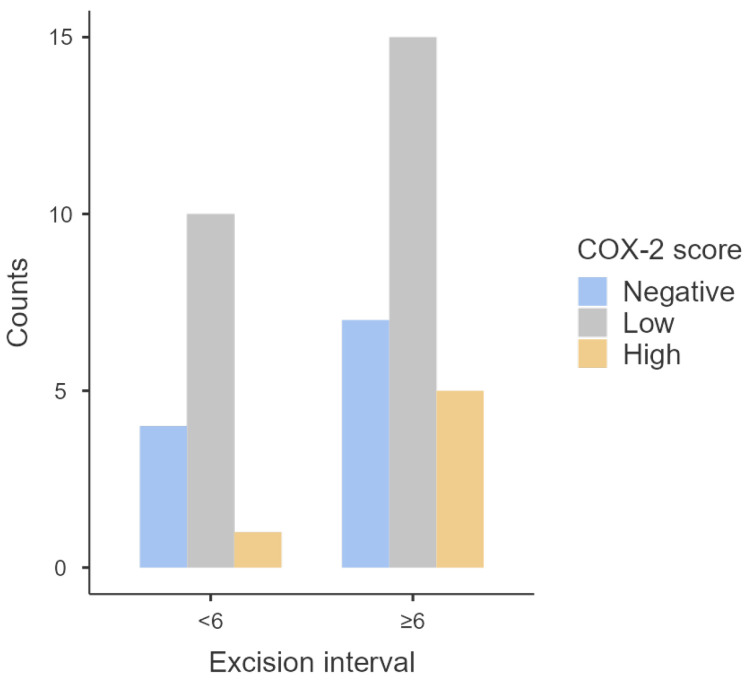
Distribution of COX-2 scores according to excision intervals.

No statistically significant association was observed between COX-2 and excision interval (*p* = 0.60), with most positive tumors maintaining low COX-2 levels across time (*n* = 25) (Table 5 and Figure 5). Furthermore, no statistically significant differences were detected in the medians of COX-2 scores between tumors excised sooner and later (*p* = 0.74).

The correlation between these two biomarkers was non-significant (*p* = 0.32) and negative (−0.16).

## 4. Discussion

The old dogma that most gray horses will inevitably develop melanomas and that this neoplasm is benign has led to some negligence in the management of this disease [13]. There is still some ignorance among owners about these tumors, the future consequences they can bring, and the importance of early monitoring for this disease [7,15]. The idea that it is normal for a gray horse to have melanomas ignores long-term animal welfare. It is therefore important to assess what changes occur in these tumors in the long term, and what differences exist between tumors excised sooner and later. This work could provide some evidence that may help veterinarians to advise owners.

One of the first results obtained in this study was that tumors excised later had significantly larger dimensions compared to tumors excised sooner, which was to be expected given the literature [7,9,13]. As time passes, tumors are 11.2 times more likely to have larger dimensions. In fact, several authors mention that the greatest damage caused by equine melanomas is secondary to the extreme expansion of the tumor mass that can occur with time and which may compress important structures [7,9,13,25]. Thus, it becomes clear that removing these tumors as soon as possible is the best clinical approach in order to avoid these problems. This approach also helps to avoid the need for advanced surgical techniques, which are not always accessible to general practitioners and may not be easily accepted by the owners due to economic constraints [7]. Although this work could not corroborate this statement, some authors are of the opinion that larger masses are prone to ulcerate, contributing to the development of secondary infections and causing pain [15,16]. Although ulceration can be caused by other factors independent of tumor size, such as self-trauma, the low number of ulcerated tumors in our sample may have been the reason for this statistical result.

In addition to an increase in tumor size, we also found that the passage of time led to an increase in the number of tumors carried by each horse, which was also reported by Knottenbelt 2018 [15]. Although some may think that a 2 cm nodule on the perianal region of a horse will not affect much the horse’s life, this scenario changes dramatically when we realize that, if left untouched, this small tumor will not only grow, but may also join to other new masses that will likely emerge with time. What we do not know is whether these new nodules will have emerged spontaneously, or if they will be metastases of previous nodules. Nevertheless, based on these results, it would be imprudent to think that these nodules do not warrant concern. It seems very unlikely that a tumor will not evolve at all over the years.

Currently, there is no association between gender and the prevalence of equine melanoma, with both females and males being equally affected [7]. Considering that some females (7/18) included in our sample were used for reproduction, and that the localizations of the tumors were the tail and the perianal region, it would be expected that these masses would be detected early during reproductive procedures. Furthermore, these masses are prone to cause some embarrassment during insemination and birth, so it is beneficial to remove them as soon as they appear [7]. This type of constraint does not apply to non-breeding mares or saddle horses, and, depending on the tumor’s size, this mass location may not interfere with their performance. Therefore, we expected that breeding mares would have these tumors removed earlier and that this would influence the statistics between genders in our sample. However, 10/18 females and 9/16 males presented melanomas for more than 6 years, and 8/18 females and 7/16 males for less than 6 years (results not shown), culminating in the absence of a significant difference.

For the reasons mentioned above, we also expected to find differences in tumor duration between breeding and non-breeding females. We expected that breeding-mares would have these tumors removed earlier. However, despite the majority of breeding mares (5/7) presenting lower excision intervals and the majority of non-breeding mares (8/11) presenting higher excision intervals (results not shown), this speculation was not confirmed in our study, probably due to the low number of breeding mares.

The fact that no association between tumor size and histological classification was found in this work is in agreement with the literature regarding canine melanoma [22], and may suggest that, from a histological point of view, a smaller tumor is not less cause for worry than a large tumor. Clinically, a larger tumor may have all the constraints previously mentioned, but nothing refutes the possibility that a smaller tumor could be highly malignant, histologically. Therefore, a therapeutical approach should bear this in mind and, once again, not underestimate these smaller tumors.

Although the retrospective nature of this work impairs our ability to discuss in detail the nursing difficulties and the impact on athletic performance associated with melanoma excision, these results do allow for some reflection. According to the literature, melanomas appear around 6–7 years of age in horses. It is understandable that there is some reluctance from owners to carry out a surgical procedure in a sensitive area, which could have some impact on their horse’s sporting performance for a certain period of time. However, looking at our results, it is better to remove these tumors in horses aged 6–7 years than when the horses are 12–14 years old (after more than 6 years of tumor duration), which, in certain sports, corresponds to the peak of their sportive career. Our results indicate that these tumors, after 6 years, have a higher probability of acquiring large dimensions, meaning that the animal may be subjected to a more invasive intervention that could bring worse consequences and a longer recovery time.

In light of our results, letting time pass is not only bad clinically, but also histologically. It seems that with time, the more likely it is that the tumor will become malignant. In our sample, tumors excised later were five times more likely to be malignant. This result makes sense given the literature, which mentions that equine melanomas have a time-dependent transition to malignancy [1], although we were not able to confirm that this transition occurred since we did not perform different biopsies of the same tumor across time. Despite acquiring the histological characteristics of malignancy that would allow these tumors to become aggressive, for some as-yet-unknown reason, the majority do not become aggressive.

Although no association was found between excision interval and tumor ulceration, once more, the low number of ulcerated masses may have influenced the results.

Another finding of this work that corroborates the opinion of some authors, is that tumor ulceration was not associated with histological malignancy [1,14]. When the tumor ulcerates, owners are often most concerned, due to the poor appearance it acquires. However, this does not mean that it is more aggressive or malignant. Regardless, an ulcerated tumor can cause the horse greater discomfort in the short term [2].

Three points addressed in this work, namely, tumor size, number of tumors, and histological classification over time, are not necessarily new, but the existing knowledge is mostly empirical, and not objective or scientifically measurable [7,15]. This work provides scientific evidence for previously acquired empirical knowledge, giving clinicians more security during their practice and conversations with owners.

Immunohistochemically, it seems that E-cadherin and COX-2 do not evolve substantially across time. In general, equine melanomas, even malignant ones, maintain high levels of E-cadherin and low levels of COX-2 across the years, differing from other species [35,45]. These tumors may not only remain dormant at the clinical level, but also at the molecular level. Given the roles of these biomarkers in the progression of cancer, the fact that they remain stable seems to be in accordance with the known biological behavior of equine melanoma. These tumors are characterized by a slow expansive growth over the years, without evidence of invasiveness or metastization. High levels of E-cadherin may contribute to the cohesion of the tumor mass, and low levels of COX-2 may contribute to the slight and slow growth pattern that occurs across the years. Therefore, some important steps in tumor dissemination and metastization, such as epithelial-to-mesenchymal transition, characterized by a loss of E-cadherin and overexpression of COX-2, may not occur. However, since we only studied the protein levels of these biomarkers, gene expression studies are necessary to corroborate this theory. Furthermore, other molecular alterations that might occur with time and that might justify the results of this work (such as the increase in tumor size and transition to malignancy) should be studied.

The main limitation of the present work is the lack of follow-up, which does not allow us to know the consequences of tumor excision and if the excised tumors recurred. However, some studies have already reported no recurrence in the long term after equine melanoma excision [20,46]. Furthermore, the small sample size and limitations of data collection could have negatively influenced the results of this work. Further prospective clinical studies that individually monitor the clinical, histological, and immunohistochemical progression of tumors in horses will be necessary in order to corroborate the findings of this retrospective study.

## 5. Conclusions

With this work, we provide scientific and objective evidence that time significantly influences equine melanomas, contributing to an increase in their size, number, and malignancy. As such, this work clarifies the importance of early intervention in preventing future complications caused by these tumors. This data may help clinicians in advising horse owners.

## Figures and Tables

**Table 1:** Relationship between excision interval and tumor size.

	Tumor Size				
Excision Interval	Small	Medium	Large	Total	* p *
** <6 years **	10	4	1	15	** 0.038 **
** ≥6 years **	10	5	12	27	
Total	20	9	13	42	

**Table 2:** Relationship between excision interval and number of tumors.

	Number of Tumors			
Excision Interval	Single	Multiple	Total	* p *
** <6 years **	5	10	15	** 0.011 **
** ≥6 years **	0	27	27	
Total	5	37	42	

**Table 3:** Relationship between excision interval and histological classification.

	Histological Classification			
Excision Interval	Benign	Malignant	Total	* p *
** <6 years **	8	7	15	** 0.035 **
** ≥6 years **	5	22	27	
Total	13	29	42	

**Table 4:** Distribution of E-cadherin score levels across different excision intervals.

	E-Cadherin Score				
Excision Interval	Negative	Low	High	Total	* p *
** <6 years **	0	8	7	15	** 0.20 **
** ≥6 years **	2	7	18	27	
Total	2	15	25	42	

**Table 5:** Distribution of COX-2 score levels across different excision intervals.

	COX-2 Score				
Excision Interval	Negative	Low	High	Total	*p*
**<6 years**	4	10	1	15	**0.60**
**≥6 years**	7	15	5	27	
Total	11	25	6	42	

## Data Availability

Data are contained within the article.
